# Non-invasive, Zip Type Skin Closure Device vs. Conventional Staples in Total Knee Arthroplasty: Which Method Holds Greater Potential for Bundled Payments?

**DOI:** 10.7759/cureus.4281

**Published:** 2019-03-20

**Authors:** Omar Alnachoukati, Roger Emerson, Muraya Muraguri

**Affiliations:** 1 Orthopaedics, Texas Health Resources, Plano, USA; 2 Orthopaedics, Texas Health Resources, Allen, USA

**Keywords:** zip closure, staples, total knee arthroplasty, bundled payments

## Abstract

Introduction: A new, noninvasive zip surgical skin closure device has recently been introduced into the market and introduces a new alternative to the traditional staple closure method which has dominated the industry for many years.

Methods: This was a retrospective chart review and case study of a consecutive series of 130 patients who underwent primary total knee arthroplasty (TKA) from April 2016 to November 2016. The first 65 patients in this series underwent a primary TKA utilizing the standard staple closure method (staple group). The next 65 patients underwent a primary TKA through the new zip surgical skin closure method (zip group). Charts were reviewed for incision-related phone calls to the clinic, additional clinic visits due to incision concerns, antibiotic prescriptions due to incision complications, and incision-related emergency room (ER) admits.

Results: The staple group had a significantly higher amount of incision-related phone calls made to the clinic as compared to the zip group (20 versus 8, P < .05, respectively). The staple group also had more incision-related ER admits, more incision-related clinic visits, and more antibiotics prescribed due to incisional complications than the zip group (1 versus 0, 5 versus 2, and 4 versus 1, respectively), although it was not proven to be significant (P > .05).

Conclusion: This is the largest patient cohort study comparing the noninvasive zip-type closure device to staple closures in TKA cases and provides insight on how a simple change in the closure methodology can lead to many potential downstream savings when considering a bundled payment model.

## Introduction

Healthcare spending in the United States is rising at an unsustainable rate. Total joint arthroplasty is the largest expense for a single condition among Medicare beneficiaries, constituting approximately 5.7% of annual Medicare expenditures [1]. Given that the annual expenditure for major joint replacements is estimated at $7 billion [2], the Centers for Medicare and Medicaid Services (CMS) recently announced its finalization of the Comprehensive Care for Joint Replacement (CCJR) model, holding hospitals responsible for the quality of care they deliver to Medicare beneficiaries undergoing hip and knee surgeries from surgery through recovery [3]. The grouping of services under a single bundled price inherently shifts the risks of overspending from the payer to the providers [4]. Providers will have to come up with novel and creative ideas to help curb costs while improving quality of care.

Staples have been used for surgical skin closures for many years, and although effective at tightly closing incisions, this invasive technique has come with its own set of complications. Conventional staples are mainly used for skin closure after total knee arthroplasty (TKA) [5-6]. Patients tend to feel pain and fear when staples are removed [5] and can suffer from infections, bleeding, and additional scar formation on piercing sites after the staples are removed [7-9]. The noninvasive zip surgical skin closure device gives the surgeons a fresh new technique for skin closure to use at their disposal. The purpose of this study is to determine the safety and effectiveness of the zip-type closure device, as compared to the traditional staple closure technique, utilized to close incisions following total knee arthroplasty. The secondary objective is to conclude on any cost saving potential this new device may hold when considering a 90-day global period, bundled payment system.

## Materials and methods

The approval of the Texas Health Resources Institutional Review Board at the local participating location was obtained for this study (#944633-1) and a waiver of informed consent was approved. There was no research funding provided by the sponsor for this study. This was a retrospective chart review and case study of a consecutive series of 130 patients who underwent primary total knee arthroplasty (TKA) from April 2016 to November 2016. Data abstraction was conducted by the study team, and thus, the data was not anonymized prior to access. The first 65 patients in this consecutive series underwent a primary TKA using the standard staple closure method (Ethicon, Cincinnati, OH, USA) (staple group). The next 65 patients underwent a primary TKA through the new zip surgical skin closure method (ZipLine Medical, Inc., Campbell, CA, USA) (zip group). The switch from staple to zip surgical skin closures was due to a transition in the surgical closure standard of the care process. All surgeries were done by the same surgeon, utilizing the same implants (Zimmer Biomet, Warsaw, IN, USA), and the closures were done by the same surgical physician’s assistant through standard procedures. Simultaneous bilateral TKA patients and revision TKA patients were excluded to maintain consistency between both groups.

TKA postoperative deep vein thrombosis (DVT) prophylaxis, unless contraindicated, consisted of patients being sent home on Coumadin for two to four weeks according to previous medical history. The Coumadin goal was the international normalized ratio (INR) value of 1.8 - 2.5. Regarding antibiotics prescribed for wound complications, the patient was started on antibiotics postop if there was increased drainage from the surgical site that was yellow, green, or foul-smelling, a non-healing area of the incision, i.e., redness/warmth around the incision, painful to touch, or a fever of 100.0° F or higher. Thromboprophylaxis and wound complication protocols remained consistent between both groups.

TKA surgical procedures were the same in both groups and conducted at the same surgical facility. For all patients, a tourniquet surrounded the thigh during the operation. All TKA’s were implanted through the medial parapatellar approach. At the final follow-up of one month, there were 65 patients each in the zip group and the staple group. There were no significant differences between age, gender, or laterality, although body mass index (BMI) was considered to be statistically significant between the two groups, with lower BMI’s favoring the staple group (Table 1).

**Table 1:** Patient Demographics of Zip Group and Staple Group Values presented as mean + standard deviation or number of cases (%). *Statistically significant BMI: body mass index

	Zip Group	Staple Group	P-value
Characteristics	(n = 65)	(n = 65)	
Age	66.8 + 8.4	68.3 + 8.2	0.288775
Gender:			
Male	23 (35.4)	25 (39.5)	0.718805
Female	42 (64.6)	40 (61.5)	
BMI	34.3 + 7.3	31.4 + 7.6	.027379*
Laterality:			
Left	32 (49.2)	27 (41.5)	0.382326
Right	33 (50.8)	38 (58.5)	

For skin closure, two methods were used sequentially: the conventional staple closure method and a zip-type, non-invasive, skin-closing method. All knee capsules were closed with Vicryl sutures and #2 Quill™, a knotless tissue-closure device (Surgical Specialties Corp., Westwood, MA, USA), the subcutaneous tissues were closed with 2-0 Vicryl, and the final skin layer was closed utilizing either the zip or the standard staple closure method. Closing the skin with staples consisted of flexing the knee at 90 degrees, pinching the skin together, and using a staple gun to seal the skin.

Utilizing the zip closure, the skin was cleaned of residue and dried, and the zip closure applied to start at one end of the incision, with the knee placed in 45-60 degrees of flexion per manufacturer recommendations. The zip was centered along the incision as it was applied. The skin was closed by pinching the skin edges together over each strap of the zip until the skin was approximated and then pulling on the strap loop to lock in the tension. This was done for each strap-in together to achieve approximation along the incision, and the excess strap length was trimmed with scissors.

Patients were followed from the time of surgery until the first postoperative visit in the clinic (postoperative day 21-28). Patients’ medical records were reviewed for any clinic phone calls relating to incisional complications, if there were any clinical visits regarding incisional concerns, if antibiotics were prescribed for any incisional complications, and for any incisional complication, such as emergency room (ER) admits. Employee pay scales, median annual pay (from payscale.com [10]), and average observed clinic time spent on individual tasks (Table 2) were used to determine the cost to the clinic (Table 3). Clinic time spent managing incision complications was used to determine opportunity cost.

**Table 2:** Average Time (Minutes) Spent Per Task in Clinic

Task	Time
Call Medical Assistant	15 minutes
Call Physician's Assistant	5 minutes
Front Desk Scheduling	5 minutes
Call Surgeon	5 minutes
Staple Removal	20 minutes Physicians Assistant, 10 minutes Medical Assistant
Clinic Visit	30 minutes Medical Assistant, 30 minutes Surgeon
Skin Check	15 minutes Medical Assistant, 15 minutes Physicians Assistant

**Table 3:** Median Employee Salary According to payscale.com [10]

Staff	Salary/Hour
Front Desk	$12.00
Medical Assistant	$14.00
Physician's Assistant	$49.00
Nurse	$31.00
Primary Care Provider	$81.00
Surgeon	$111.00

A follow-up clinic visit was calculated as $300, and a new patient visit was calculated as $500 (typical charge values for these visits, not to be confused with reimbursed values, as that is highly variable depending on the insurance). Clinic visits requiring a medical assistant and a physician’s assistant were considered the equivalent of a follow-up visit. Clinic visits requiring a medical assistant and a surgeon was considered the equivalent of a new patient visit.

Students T-tests were used to compare results between the zip group and the staple group. P values < .05 were considered statistically significant. Statistical analysis and graphs were prepared using Microsoft Excel (Microsoft Corp., Redmond, WA, USA) macros.

## Results

**Figure 1:**
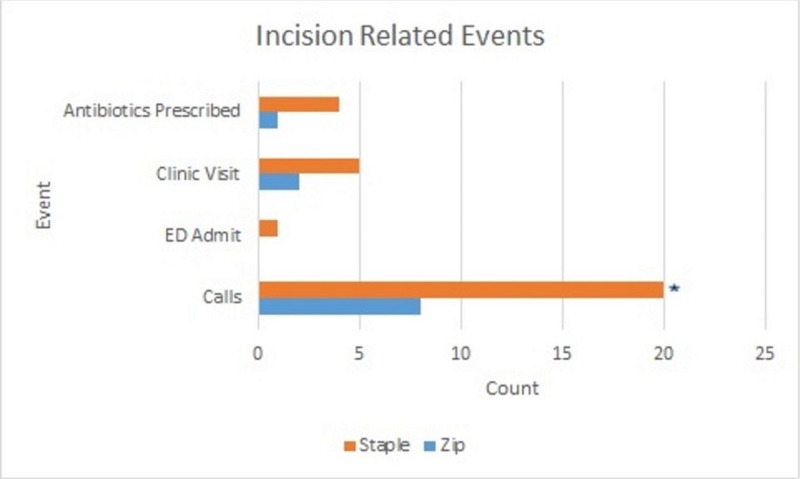
Incision-related events Cumulative total number of incidences per incision-related event; calls to the clinic, Emergency Department (ED) admits, additional clinic visits, and antibiotics prescribed. *Significance between two groups (P < .05)

The staple group had a significantly higher amount of incision-related phone calls made to the clinic as compared to the zip group (20 versus 8, P < .05, respectively). The staple group also had more incision-related ER admits, more incision-related clinic visits, and more antibiotics prescribed due to incisional complications than the zip group (1 versus 0, 5 versus 2, and 4 versus 1, respectively), although not proven to be significant (P > .05) (Figure 1). Of note, three (5%) of the 65 zip patients reported the ability to remove the closure device themselves. At the final follow-up of one month, there were 65 patients each in the zip and the staple groups.

One patient in the staple group developed an infection, erythema, and swelling around the knee incision with slight drainage. The patient was transferred to the ED from a rehabilitation center 10 days postop. One patient in the zip group reported falling directly on their operative knee after being discharged, causing dehiscence, which required surgical debridement of the dehisced area and re-closure of the surgical site. This patient was not considered a surgical site infection-related ER admit, as the patient had a traumatic fall on their operative knee, causing the incision to physically break open.

**Figure 2:**
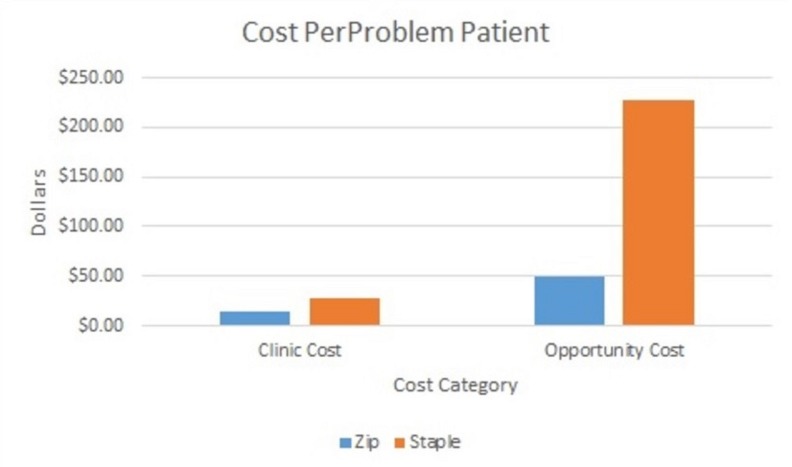
Cost per problem patient Clinic cost was determined by the time spent during clinic tasks managing problem patients (Table 2) and multiplying by employee salary (Table 1). Opportunity cost per problem patient was calculated by determining equivalents to time spent managing problem patients with new patient/follow-up patient visits. Thirty minutes of medical assistant (MA) time and 30 minutes of physician's assistant (PA) time equal a follow-up visit ($300 billable visit). Thirty minutes of MA time and 30 minutes of surgeon time equal a new patient visit ($500 billable visit).

There were seven patients who had incision-related problems in the staple group (BMI: 33 + 10.3) as compared to four patients in the zip group (BMI: 46 + .6). Problems reported in the staple group included redness or hardness around the incision, swelling, inflammation, infection, and the incision looking “open”. Problems reported with the zip group included redness, the closure device coming partially undone, drainage, blisters near the incision site, and red spots of granulation tissue. The clinic cost per problem patient was almost double in the staple group in comparison to the zip group ($27 versus $14.75, respectively) and opportunity cost calculated per staple problem patient was more than quadrupled when compared to the zip group ($228 versus $50, respectively) (Figure 2).

## Discussion

Healthcare spending has increased substantially and the CMS projects health spending to continue to rise to 20.1% of gross domestic product (GDP) by 2025 [3]. With the introduction of the bundled payment system to help curb costs, the risk has shifted from the payer to the providers [4], immediately incentivizing physicians to provide higher quality care at a lower cost or suffer financial consequences [11-12]. CCJR now places all risk for the 90-day episode with the hospital [2]. Providers must initiate a proactive approach to the entire total joint arthroplasty process and come up with new and innovative ideas to help curb costs and increase satisfaction, essentially increasing the overall value of care. This study examines the period of transition from the staple to zip skin closure methods as the preferred method of closure for the standard of care and seeks to compare the two methods in an effort to determine any benefits.

Staples are mainly used for skin closure after TKA [5-6]. However, patients tend to feel pain and fear when staples are removed [5], while also suffering from infections, bleeding, additional dressings, or scar formation on piercing sites after staples are removed [7-9]. It is suggested that the non-invasive character of surgical skin closure may reduce the risk of infection [13]. Our series further confirms these studies as the zip group had a 75% reduction in the prescription of antibiotics when compared to the staple group.

**Figure 3:**
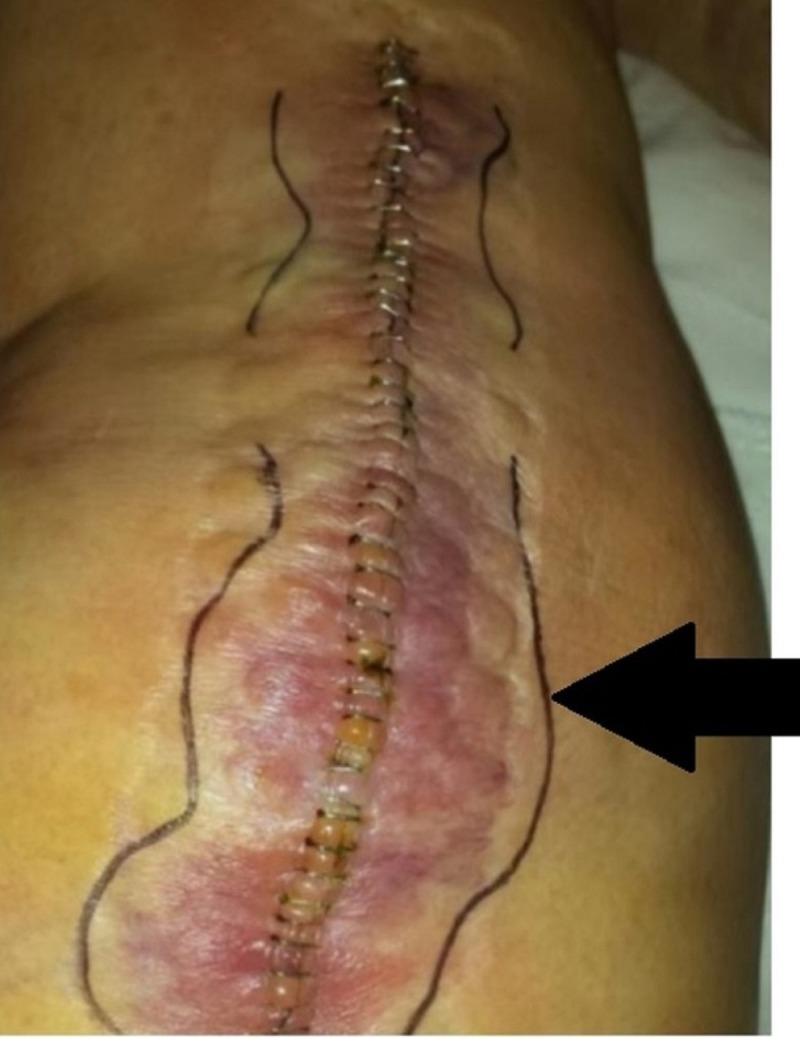
Picture showing a staple closure patient who was readmitted to the hospital for a postoperative wound infection

Patient satisfaction and perception are important factors to consider when discharging post-surgery, and skin management has also become an integral factor for determining patient satisfaction [14]. If a patient or caregiver is concerned, they may be readmitted even though a hospital admission is not necessary. The lone skin-related readmission in this study was attributed to a suspected surgical site infection in the staple group (Figure 3).

McCormick et al. reported the three main causes of readmissions were skin complications, surgical site infections (SSIs), and medical issues [15-17]. Looking exclusively at direct costs (implant costs, hospital room and board, medications, and medical supplies), the mean cost of TKA readmissions is $13,008 [18]. Readmissions have been shown to significantly impact the cost-effectiveness of bundles and often represent complications which need to be minimized to improve quality [19]. Reducing the occurrence of these “bundle busters” will not only help reduce risk to the providers from a cost perspective but can ultimately improve the quality of care for patients, inherently increasing the overall value of care.

Patient phone calls to the clinic can put a serious strain on resources and the perception of patient satisfaction. Calls are often required to be answered within the same day, and simply returning a concerned patient’s call can turn into a drawn-out session of phone calls and voicemails. Incorporating the zip closure system in TKAs has significantly reduced the number of calls received by the clinic from patients with incision-related concerns, which has a direct savings effect with regards to clinic productivity and theoretically increases patient satisfaction.

The zip group demonstrated a 60% reduction in incision-related calls to the clinic during the course of this study. Many clinics are rated based on patient surveys, thus improving patient interaction and satisfaction which can have a dramatic effect on clinic rating systems. With the evolution of value-based care comes the demand for better quality treatments. Today’s healthcare patients are well-informed, knowledgeable, and seeking new treatment options that can provide heightened benefits. Ko et al. found that the Vancouver scar score for the cosmetic outcome on postoperative day 90 was significantly better in the zip group compared to that of the staple group (4.6 + 0.7 versus 6.9 + 1.3, P < 0.05, respectively) [20]. The patient visual analog scale (VAS) pain scores were also shown to be significantly lower on postoperative days 1, 3, and 14 in comparison to the staple closure group. Lower pain and cosmetically more appealing scars can make the difference in TKA patient-perceived outcomes from one surgery center to the next.

**Figure 4:**
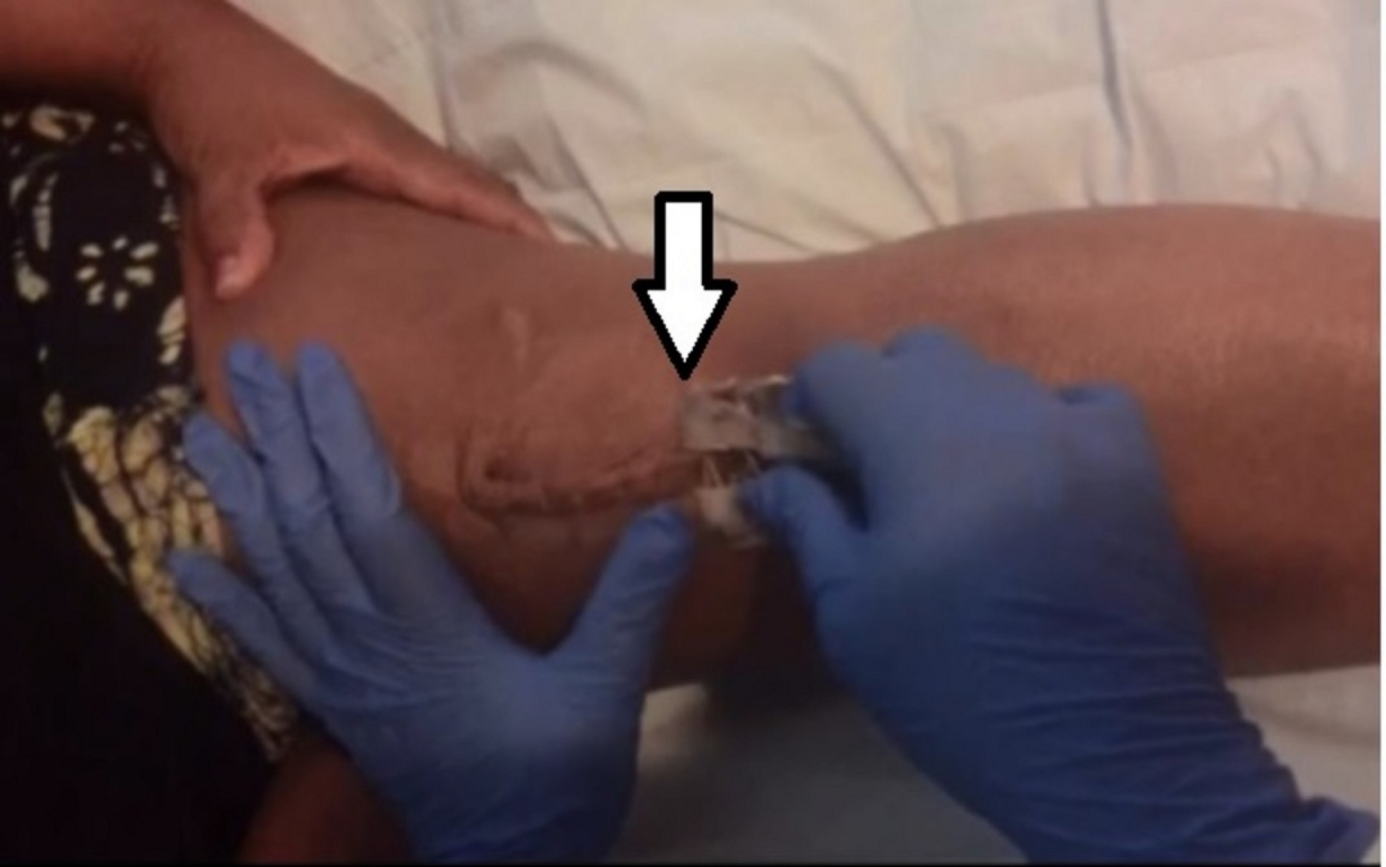
Picture of ZipLine removal

Three patients in the zip group reported the ability to remove their own closure devices safely and effectively. Removal of the device is similar to removing an adhesive bandage (Figure 4). Staple closures require a healthcare professional to remove the staples postoperatively, either by a home health visit or a clinic visit.

Three patients in the staple group came into the clinic for staple removal. Time spent removing staples in the clinic could have been used to follow-up on surgical patients, which would generate more revenue for the clinic considering staple removal visits are included in the global period and are not billable. Post-acute care and rehabilitation facility costs account for over 50% of inpatient facility costs for TKA patients [11]; thus, having the ability to reduce or even eliminate these postoperative services holds a greater savings potential for bundled providers. Joint classes may be an effective way to train TKA patients on how to remove their own zip-skin closures in the future, placing less reliance on home health agencies to manage surgical incisions postoperatively. Two patients in the staple group came in for an additional clinic visit to verify that their incision was satisfactory, one of which the surgeon suspected a reaction to the staples was the cause for the redness and inflammation. One patient in the zip group came into the clinic on two separate occasions for a skin check and was suspected to have cellulitis. All of these patients were placed on a round of antibiotics for one week. Surgeons are by far the most valuable resource in the healthcare setting. With limited time, yet an enormous responsibility for patient well-being, practicing a more preventative approach toward surgical wound care can help use these providers' time more efficiently. Surgeons' time used for wound checks or incision-related ER admits not only places a financial strain on the healthcare system but also slows the continuum of care, further delaying potential surgical patients from being seen and scheduling surgery.

**Figure 5:**
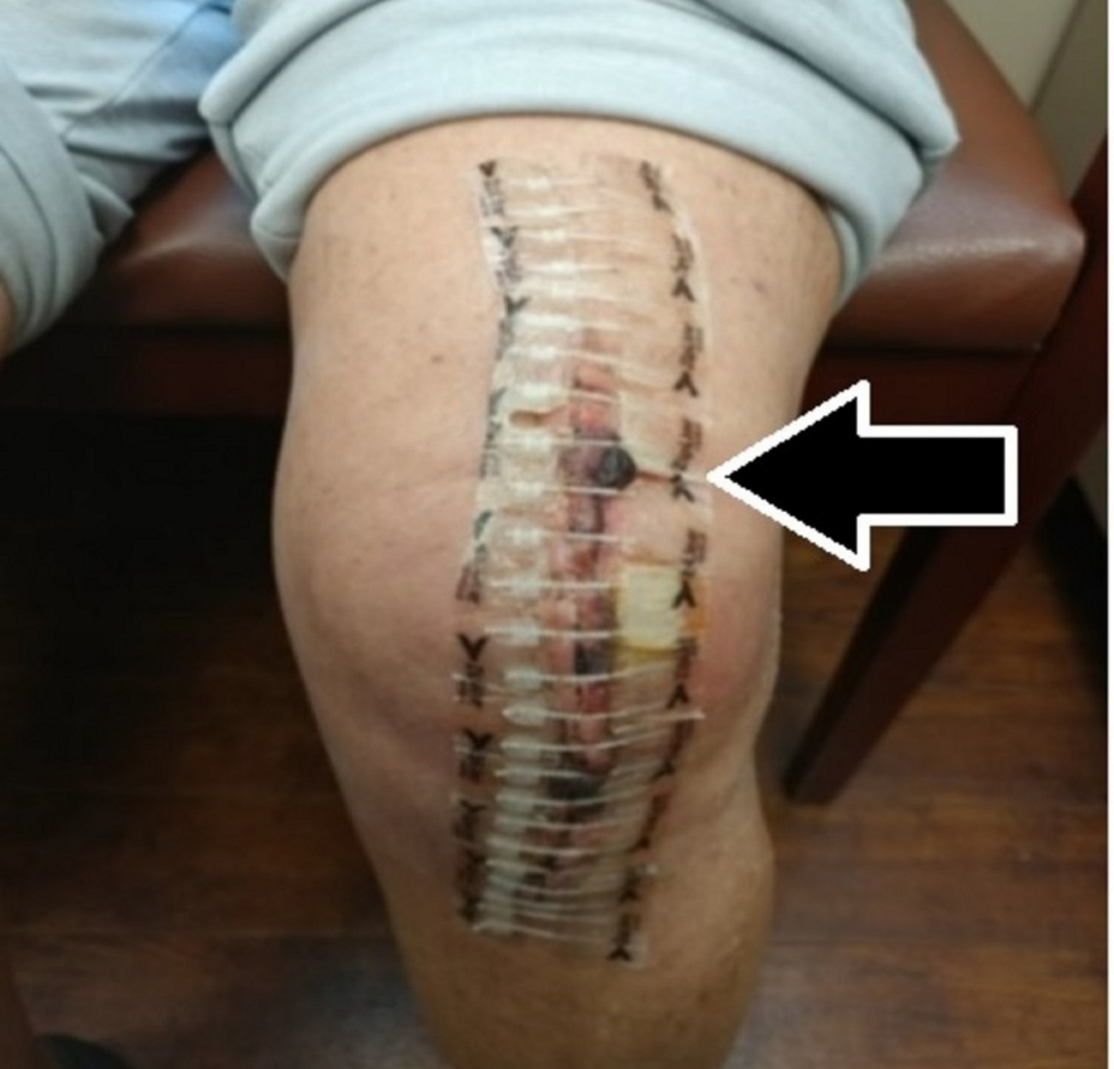
Picture of ZipLine removal. Picture showing patient with zip-type closure who developed blisters around the incision.

A superior feature of the Zip surgical skin closure device is the ability to adjust its length and tension [21]. This feature helps further customize the closure per patient, moving away from the one size fits all approach of staples. When approximating zip closure tension, it is encouraged to anticipate swelling of the knee and resist over-tightening the closure. Over-tightening the zip closure could lead to the formation of blisters. Of note, only two zip patients (3%) reported blister formation around the incision, which did not progress to any delayed incision healing (Figure 5).

One patient reported that the zip closure started coming off at the upper left side of the incision on postoperative day 4. The device was reinforced with Steri-Strips™ (3M, St. Paul, MN) and the incision healed normally. Keeping the adhesive portion of the zip closure away from the base of the incision is critical to sustaining adhesion to the skin. Surgeons should be cautious of patients with adhesive allergies or highly sensitive skin, as the adhesive component of the zip closure may cause reactions, such as a rash or blisters on the skin. Also, given that the average BMI for problem patients in the zip group was 46 (although not all patients with high BMI’s had problems), it is recommended to practice caution when using the non-invasive closure in patients with a BMI of 45 or greater. Patients with very thin skin may require other closure methods.

**Figure 6:**
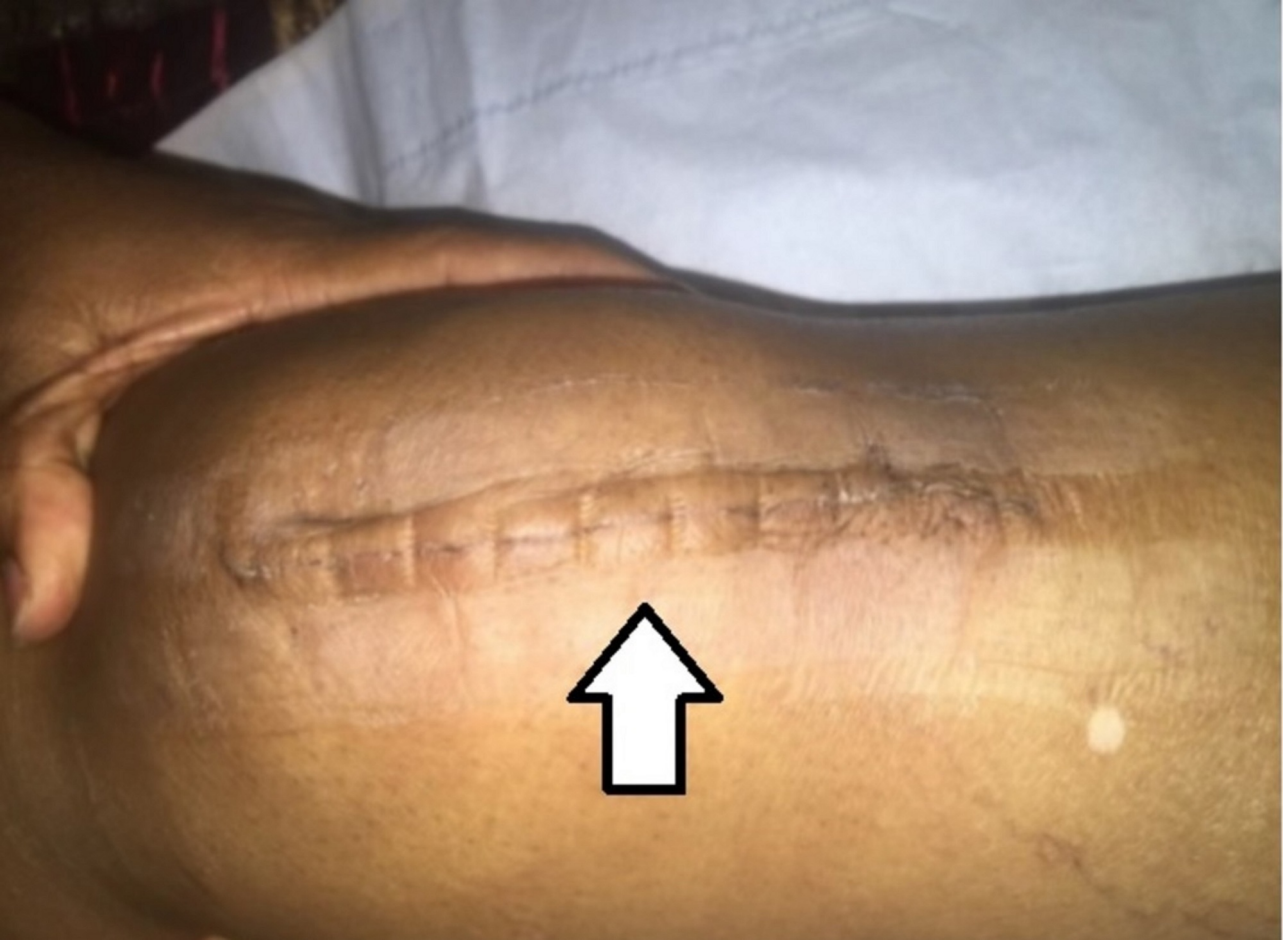
Close-up picture of zip closure scar at day 14 postop.

When applied appropriately, the zip closure has the ability to generate cosmetically appealing scarring (Figure 6), which can improve patient satisfaction.

Considering the variability of medical device pricing, the ZipLine closure is generally more expensive than conventional staples on a per unit cost basis ($85 vs $33, respectively), although given the various avenues of cost-saving potential and increased patient benefits discussed throughout this study, one can surmise the superiority far outweighs the additional upfront cost. Ultimately, it is up to healthcare executives to conduct their own cost analysis and determine if an increase in upfront costs is worth reaping the potential downstream benefits.

The retrospective component of this study may serve as a limitation, although patients were prospectively followed. Further evaluation with randomization of the two groups is recommended. Another limitation is the significant difference between the groups with regards to BMI; granted that higher BMI in the zip group was less favorable, outcomes still showed superiority over the staple group. Ultimately, the authors decided to retain the consecutive series aspect of the study over selectively eliminating patients to balance out BMI between the two groups. The scope of this study focuses primarily on the clinic setting, but one must also consider the hospital and postoperative care units collectively when determining bundled payment program components and their inherent benefits. Personnel and resource utilization may vary among institutions; therefore, any efficiencies described in this study must be considered on a case-by-case basis. A power analysis was not conducted, and given the low incidence of complications across the study, statistical significance will be difficult to substantiate. Randomized studies with larger patient sample sizes are needed to solidify conclusions more robustly, although this study can surely act as a pilot for further investigation. The strengths of this study include the relatively high volume of TKA patients coupled with having access to an electronic medical record system (Epic, Verona, WI) that interfaces with the clinic, hospital, and postoperative care units.

## Conclusions

In conclusion, this is the largest patient cohort study we are aware of that compares the noninvasive zip-type closure device to staples in TKA cases and provides insight on how a simple change in the closure method can lead to many potential downstream savings when considering a bundled payment model. A decrease in patient incision-related phone calls, a decrease in incision-related antibiotic prescription, a decrease in incision-related clinic visits, reduction in incision-related ER visits, a reduction in the number of incision-related problem patients, and a reduction in cost per problem patient were all benefits observed in the zip closure group in comparison to the staple closure group. Given the benefits of the zip-type closure, surgeons and healthcare executives, especially those participating in bundled payment systems, may consider using the zip-type skin-closing device for patients undergoing TKA in an effort to improve efficiency, quality, and value of care.
